# Executive functioning moderates the decline of retrieval fluency in time

**DOI:** 10.1007/s00426-022-01680-0

**Published:** 2022-04-25

**Authors:** Drahomír Michalko, Martin Marko, Igor Riečanský

**Affiliations:** 1 Department of Behavioural Neuroscience, Institute of Normal and Pathological Physiology, Centre of Experimental Medicine, Slovak Academy of Sciences, Sienkiewiczova 1, 813 71 Bratislava, Slovakia; 2 Department of Applied Informatics, Faculty of Mathematics, Physics and Informatics, Comenius University in Bratislava, Mlynská Dolina F1, 842 48 Bratislava, Slovakia; 3 Social, Cognitive and Affective Neuroscience Unit, Department of Cognition, Emotion, and Methods in Psychology, Faculty of Psychology, University of Vienna, Liebiggasse 5, 1010 Vienna, Austria; 4 Department of Psychiatry, Faculty of Medicine, Slovak Medical University in Bratislava, Limbová 12, 833 03 Bratislava, Slovakia

## Abstract

Prevailing theoretical accounts consider that automatic and controlled processes are uniformly engaged in memory retrieval across performance of the semantic verbal fluency (SVF) task. We tested this proposal against the alternative, namely that a rapid automatic retrieval, exploiting stable associative structure in the early stages of the performance, is followed by a slower, more executively demanding, retrieval in later stages. Eighty-five healthy adults completed low- and high-demand SVF tasks that were assessed for retrieval rate, response typicality, and inter-response similarity across the performance. Additional measures of executive functioning were collected to estimate individual differences in executive control. We found that decrease in fluency in time was associated with lower typicality and weaker semantic similarity of the responses. Critically, the time-dependent retrieval slowing was steeper in individuals with less efficient interference control, particularly in high-demand SVF tasks. Steeper retrieval slowing was also associated with poorer working-memory capacity. Our findings show that the relative contribution of automatic and controlled processes to semantic retrieval changes with associative sparsity over time and across task demands, and provide implications for the use of SVF tasks in clinical assessment.

**Supplementary Information:**

The online version contains supplementary material available at 10.1007/s00426-022-01680-0.

## Introduction

Semantic cognition supports adaptive retrieval of knowledge, which is traditionally assessed by semantic verbal fluency (SVF) tasks requiring an individual to retrieve exemplars of a given semantic category over a limited time. Studies that have employed SVF tasks in healthy individuals, as well as patients with neuropsychiatric disorders, have indicated that semantic retrieval is governed by an interplay between two functionally complementary systems that subserve automatic and controlled (executive) semantic retrieval, respectively (Chiou et al., 2018; Hirshorn & Thompson-Schill, 2006). Contemporary literature on semantic retrieval is vastly informative about the roles of automatic and controlled processes in the SVF, yet it fails to fully delineate their relative engagement across the task performance. The automatic retrieval is thought to act upon the semantic (or associative) links between the respective bits of knowledge (i.e., category exemplars) stored in the semantic memory with stronger links (representing higher semantic or associative similarity) enabling a more rapid spread of semantic activation from one exemplar to the other (Kumar, 2021; Mayr & Kliegl, 2000). The controlled retrieval, on the other hand, is mediated by limited executive capacities (both domain-general and -specific) that flexibly and strategically regulate the spread of semantic activation to provide a broader exploration of semantic space (Hills et al., 2013; Mayr, 2002; Troyer et al., 1997). For example, controlled semantic switching shifts the retrieval from one subcategory of closely linked exemplars (e.g., pets in the animals category) to the other (e.g., insects) when the former has been depleted (Hirshorn & Thompson-Schill, 2006; Tröster et al., 1998; Troyer et al., 1997, 1998). Regarding the control of semantic retrieval, several studies have also highlighted the contribution of interference control, inhibition, and working memory, which monitor the ongoing task performance for intrusions (Collette et al., 2001; Henry & Phillips, 2006; Rosen & Engle, 1997), maintain, and update retrieval cues to prevent fixations on a particular set of category exemplars (Hills et al., 2013, 2015; Unsworth et al., 2013). Furthermore, it has been shown that working memory capacity predicts SVF task performance (Unsworth & Engle, 2007; Unsworth et al., 2011) and that imposing a load on working memory impairs performance in the SVF task (Rende et al., 2002). Research studies by Katzev et al. (2013) and Hoffman (2018) additionally suggest that semantic control might aid retrieval in the SVF task by activating weakly associated or less typical category exemplars. Taken together, these lines of evidence concur that several control processes may facilitate retrieval in the SVF tasks.

However, little attention has yet been directed to the possibility that the contribution of the control processes in the SVF task might depend on the amount of time spent performing the task, i.e., task duration. It is a common observation that the responses in the SVF task progressively (1) take longer to be retrieved (Broday-Dvir & Malach, 2021; Crowe, 1998; Marko et al., 2019a; Marko et al., 2019b), (2) are less typical for a given category, and (3) show lower mutual semantic similarity (Beaty & Silvia, 2012; Hills et al., 2015; Thompson & Kello, 2014). Consequently, as SVF performance proceeds to less typical and semantically less similar category exemplars, several executive capacities may help to sustain fluent retrieval. Specifically, as highly typical category exemplars are getting continually depleted throughout the performance, their strong potency creates competitiveness (interference) among later retrieval goals. Interference control may be then required to efficiently resolve this gradually intensifying competition and to facilitate later SVF performance (Henry & Phillips, 2006). Working memory could aid later retrieval by efficient utilization of retrieval cues to strategically navigate the retrieval process to less accessible exemplars (Hills, et al., 2015; Unsworth et al., 2013). Further, controlled semantic search may be necessary to bias the semantic activation away from strongly towards weakly represented category exemplars occurring in later retrieval positions (Badre, et al., 2005; Marko et al., 2019a; Marko et al., 2019b). Interestingly, several studies found that decrements in retrieval rate over time are predictive of mild cognitive impairment associated with compromised executive capacities (Demetriou & Holtzer, 2017; Linz et al., 2019; Tröger et al., 2019). Moreover, this shift from automatic to controlled processing might likely occur more rapidly for high-demand SVF task categories that provide a more restricted and sparsely organized set of available category exemplars than low-demand categories characterized by a richer and well-organized pool of retrieval candidates (Katzev, et al., 2013; Mayr & Kliegl, 2000).

Therefore, we hypothesize that earlier exploitation of highly typical and strongly semantically related category exemplars decreases the availability of subsequent retrieval candidates, prompting a gradual shift from relatively automatic towards a more executively demanding semantic retrieval, especially in high-demand SVF task categories. To test this prediction, we utilized an individual-differences approach in which a group of healthy participants underwent testing on SVF tasks of low and high demands, and tasks testing domain-general and semantic executive functioning. In the SVF task, we measured how the inter-response time (IRT), response typicality (TYP), and inter-response similarity (IRS) change as a function of the response retrieval order. These observations served to assess the core assumptions that (1) IRT increases (or retrieval fluency decreases) while TYP and IRS decrease across the SVF performance, especially when SVF task demands are high and (2) that the decreases in TYP and IRS mediate the increase in IRT across the SVF performance (i.e., slowing of retrieval over time is attributable to lower typicality and weaker semantic inter-connectedness of category exemplars). Finally, we tested the hypothesis that individual differences in executive functioning moderate the retrieval slowing (increasing IRT) across the SVF performance such that the gradual slowing is inversely related to executive capacity and evident particularly under the high SVF task demands.

## Methods

### Participants

An a priori power analysis performed in G*Power (version 3.1) indicated that a sample of *N* = 85 individuals was required to detect an effect size of *r* > 0.30 (α = 0.05 and β = 0.85). Following this estimate, a group of 85 monolingual native-speaking participants (50.6% females) with an average age of 25.8 ± 4.35 years was recruited to take part in the study. Three participants had to be replaced as they produced an insufficient number of responses (two individuals) or misapprehended the instructions (one individual). No participant reported a history of severe head trauma or brain injury, neurological or psychiatric illness, or current use of pharmacological medication. The research was conducted following the Declaration of Helsinki (World Medical Association, 2013). All participants signed an informed consent form and received a financial reward after the completion of the testing session.

### Testing procedure

Participants completed a semantic verbal fluency task and four executive control tasks in random order using a computer. Stroop task and Backward digit span task assessed domain-general executive functioning, whereas a Semantic control task and an Associative chain test assessed executive control of semantic retrieval.

### Semantic verbal fluency task

A computerized version of the SVF task was used, in which participants were asked to type as many and rapidly as possible unique (i.e., not repeating) category exemplars as they came to their minds. The task included a short practice trial lasting 1 min (Plants category) and four test trials lasting 2.5 min, each administered in random order separated by rest periods lasting 20 s. Based on the studies by Mayr and Kliegl (2000) and Katzev et al. (2013), we manipulated the SVF task demands by selecting four categories of different exemplar accessibility ranging from low (Animals) through moderate (Vegetables) and moderate-high (Tools) to high (Liquids) demands category. The exemplar accessibility is defined by the expected number of unique responses, their normative frequency, and typicality. While low-demand categories include highly accessible exemplars with relatively high normative frequency and typicality, high-demand categories yield fewer responses with relatively lower normative frequency and typicality. In addition, exemplar accessibility is thought to reflect the semantic structure of the category, which can either facilitate (highly organized semantic structure) or hamper (poorly organized semantic structure) the retrieval (Katzev et al., 2013; Mayr & Kliegl, 2000). Each response in the SVF task was assessed for three measures: (1) inter-response time (IRT), calculated as the time between the execution of the response (pressing “return” key) and the first keystroke when typing the next response; (2) response typicality (TYP), calculated as the logarithm of the absolute number of occurrences of a particular category exemplar among all responses delivered within a given category in our study; (3) inter-response similarity (IRS), expressed as the logDice score (acquired from the Slovak national corpus database, http://korpus.juls.savba.sk) between the current and the previous response. The logDice computes word-to-word similarity as a logarithmic ratio of the joint occurrences of words X and Y in a specified span (five words on each side) relative to their respective occurrences in the corpus (Rychlý, 2008). To track the changes in IRT, TYP, and IRS across the performance in the SVF task, we denoted each response with its retrieval order in individual retrieval sequences calculated as the current position of the word in a word sequence divided by the length of that sequence (e.g., 5th response in a sequence of length 20 would have a retrieval order of 0.25). Note that removing erroneous entries (see response evaluation rules in Supplementary material) did not affect assigned retrieval order and calculation of target variables. Finally, participants did not see their previous entries on the screen while performing the task.

### Stroop task

The color-word Stroop task was used to assess interference control. Participants indicated the ink color of the words (yellow, blue, red, green) by pressing one of the four pre-learned keyboard buttons to 48 congruent (e.g., BLUE in blue color), and 48 incongruent (e.g., BLUE in red color) trials. Responses were assessed for reaction times (RT). Stroop interference was calculated as the RT difference between incongruent and congruent trials.

### Backward digit span task

The backward digit span task was used to estimate individual working memory capacity (WMC). Participants were asked to remember a series of sequentially displayed random numbers (0–9) and recall them in reverse order at the end of each trial. Participants were not allowed to rehearse the numbers out loud or by whispering. The starting span was two digits. After each successful recall, the span of the next trial increased by one, up to the maximum of nine digits. After each incorrect recall, the span for the next trial decreased by one. The test terminated after a total of six errors. Then, the overall WMC was calculated as the average span of the six incorrect trials minus 1.

### Semantic control task (SCT)

In the SCT, participants were presented with a cue word and tasked to choose a target word from two alternatives. Two distinct conditions varied in their demands on strategic semantic retrieval and post-retrieval selection, respectively (Badre et al., 2005; Hoffman, 2018). In the global condition, the task was to select the target word that was either strongly (low-demand trial; e.g., Bottle → Wine|Point) or weakly (high-demand trial; e.g., Sieve → River|Dispute) semantically related to the cue instead of an unrelated distractor. In the feature condition, the task was to focus on a specific physical feature (color, shape, size, or texture) and select a target word that matches the cue regarding that feature. In this condition, the cue was either strongly semantically related to the feature-matching target but semantically unrelated to the non-matching distractor (low-demand—congruent trial; e.g., [shape] Tire → Wheel|Tent) or it was semantically unrelated to the feature-matching target but strongly semantically related to the non-matching distractor (high demand—incongruent trial; e.g., [color] Ruby → Strawberry|Diamond). Participants completed 31 trials for each type of condition (global and feature) and demand (low and high), resulting in a total of 124 trials. Global and feature conditions were presented in eight separate and regularly alternating blocks (four for each condition), always beginning with the global condition block. Each block contained an equal number of low- and high-demand trials. Trials within the respective blocks, as well as the blocks themselves, were presented in random order. Trials were assessed for RT. The difference in averaged RTs to Strong versus Weak, and Congruent versus Incongruent trials, indexed controlled search cost and selective demands cost measures, respectively.

### Associative chain test (ACT)

In the ACT (Marko et al., 2019a; Marko et al., 2019b; Marko & Riečansky, 2021), participants continuously produced word chains of fixed length according to specific conditions. In the associative retrieval condition, participants produced a chain of words so that each word in the chain was semantically related to the immediately preceding word (e.g., Kitchen [starting word] ← Food ← Meat ← Body ← Part). Participants were encouraged to respond with the first association that came to their mind, which has been considered to involve automatic (i.e., spontaneous, bottom-up) semantic processing with little executive effort (Marko et al., 2019a; Marko et al., 2019b; Marron et al., 2020). In the dissociative retrieval condition, participants produced a chain of semantically unrelated words, where each new word was not related to the previous one (e.g., Fork [starting word] ← Cloud ← Tea ← Shovel ← Piano) and were reminded that delivering a related word would count as an error. Importantly, retrieving unrelated words has been repeatedly shown to require more time than retrieving associates, which has been previously attributed to additional controlled (i.e., effortful, top–down) inhibitory demands, i.e., inhibition cost (Allen et al., 2008; Collette et al., 2001; Marko et al., 2019a; Marko et al., 2019b). Whereas the retrieval type in the foregoing two conditions was fixed (i.e., the same throughout the respective chains), the third associative–dissociative condition required participants to alternate between delivering associations and unrelated words (i.e., switching between the two rules after each response; e.g., Car [starting word] ← Engine ← Forecast ← Weather ← Rope). Notably, this second manipulation is considered to engage additional resources for flexible switching between semantic sets, i.e., switching cost (Marko et al., 2019a; Marko et al., 2019b). ACT included a total of 100 trials, i.e., 25 trials for each combination of the two factors (retrieval type: associative versus dissociative × chain type: fixed versus switching) that were presented in a fixed order (fixed-associative, fixed-dissociative, alternating chain type). The responses were assessed on RT. Two measures reflecting controlled semantic processing were derived for each participant: the difference between the dissociative versus associative RTs in fixed chains indicated inhibition cost, and the difference between the dissociative RT in the alternating versus fixed chains indicated switching cost.

### Statistical analysis

The data were analyzed in R (R Core Team, 2020) and JASP (Version 0.14.1; JASP Team, 2020). Please refer to the Supplementary material for a detailed description of the data processing. Linear mixed-effect models (LMEMs) were computed to account for the measurements nested within participants by estimating a random intercept for each participant. The models were fitted using an unstructured covariance matrix and restricted maximum likelihood (REML), and *p* values were derived with Satterthwaite approximation for degrees of freedom, as these were shown to produce optimal estimates even for smaller samples (Luke, 2017). Post hoc *p* values for the comparisons of the slopes in LMEMs were corrected with Tukey HSD adjustment. Before the executive control measures were derived from the respective tasks (e.g., Stroop interference), the expected contrasts were evaluated using paired t-tests or LMEMs. Then, to evaluate the assumptions, IRT, TYP, and IRS were included in separate LMEMs (lme4 R package; Bates et al., 2015) and modeled by retrieval order, task demands (animals, vegetables, tools, liquids) and their interaction. The third assumption was assessed by a LMEM that modeled the changes in IRT as a function of the retrieval order, task demands, and their interaction (as above), but also included either TYP or IRS as a covariate (i.e., expected mediator). Before evaluating the expected mediation using this model, we checked the pre-requisite associations between TYP/IRS (mediating variables) and IRT (outcome variable) using robust percentage-bend correlation analysis (20% bending constant; WRS2 R package; Mair & Wilcox, 2020). Note that such mediation analyses (mediation R package; Tingley et al., 2014) were also inspected separately for each SVF task category for exploratory purposes. Finally, the main hypothesis that executive control moderates the slowing of IRT across the performance in SVF task, particularly in less organized (more demanding) categories, was evaluated separately for each executive control variable using LMEM. In these models, the dependent variable (IRT) was modeled as a function of a three-way interaction between the retrieval order, task demands, and executive control measure included as a moderator. Moderation effects (retrieval order × executive control measure) were also tested for each category separately. In the case of significant moderation, we reported the conditional slopes of the predictor (retrieval order) at three levels of the moderator (-1 SD, mean, and + 1 SD from the mean) denoted as low, moderate, and high, respectively. The levels of the moderator refer to the specific values from the moderator’s distribution that entered the equation testing the effect of the predictor on the outcome variable. The corresponding *p* values for the moderation effects in individual SVF task categories were adjusted by Holm–Bonferroni correction within respective executive control moderation models (i.e., six sets of four *p* values were corrected separately).

## Results

### Cognitive performance

Out of all responses, 5.40% were discarded as inappropriate, 0.44% as extremely slow (IRT > 35 s), and 2.01% as repetitions. A one-way repeated measures ANOVA indicated the expected effect of the SVF task demands on the number of retrieved category exemplars, *F*(3, 252) = 393.99, *p* < 0.001, η^2^ = 0.824. Participants retrieved on average [± 95% CI] 36.1 [34.4; 37.9] exemplars in the low-demand—Animals category, 20.0 [19.0; 21.1] in the moderate demands—Vegetables category, 17.9 [16.8; 18.9] in the moderate-high demands—Tools category, and 16.1 [15.1; 17.2] in the high demands—Liquids category, with all differences being pairwise significant (*p*_Holm_ ≤ 0.001). In addition, higher task demands were associated with a higher proportion of intrusions (i.e., errors, synonyms, repetitions) across response sequences (see Supplementary material for more information). 

Please refer to Supplementary material for the full report (including mean differences with CIs) of the comparisons related to executive control tasks. We observed a large and significant interference effect in the Stroop task, which was indicated by significantly longer RTs in incongruent compared to congruent trials, *t*(84) = 14.32, *p* < 0.001, Cohen’s *d* = 1.55. In the SCT, a significant controlled search cost was indicated by robustly longer RTs to weakly versus strongly semantically related trials in the Global condition, *t*(84) = 46.33, *p* < 0.001, Cohen’s *d* = 5.03. Similarly, longer RTs to incongruent versus congruent trials in the SCT Feature condition showed a significant selective demands cost, *t*(84) = 14.32, *p* < 0.001, Cohen’s *d* = 1.55. Finally, in the ACT, significantly longer RTs in dissociative compared to associative chains revealed a robust inhibition cost, *t*(8005) = 16.94, *p* < 0.001, Cohen’s *d* = 1.35. The retrieval type and chain type factors produced a significant interaction, *F*(1, 8003) = 49.60, *p* < 0.001. Subsequent pairwise comparisons showed that the associative RT in the alternating chain was not significantly different from the associative RT in the fixed chain, *t*(8003) = 1.25, *p* = 0.596, but that the dissociative RT in the alternating chain was significantly longer than dissociative RT in the fixed chain, confirming a significant switching cost, *t*(8004) = 10.93, *p* < 0.001, Cohen’s *d* = 1.21. Taken together, the RTs from all tasks showed the expected patterns, supporting the reliability of the cognitive indices derived from them and used in the subsequent analyses.

### Effect of retrieval order and SVF task demands on inter-response time

**Fig. 1:**
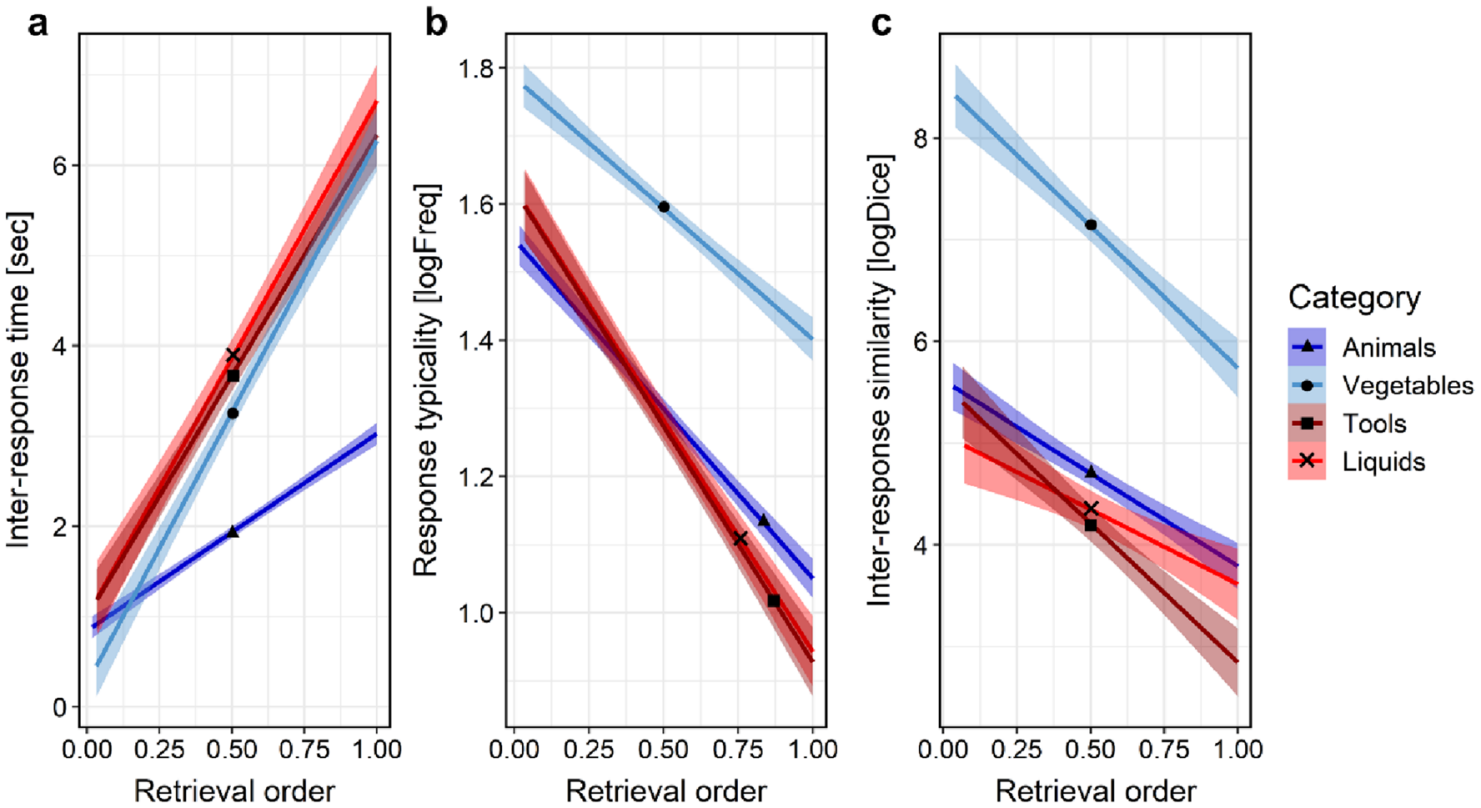
Inter-response time (**a**), response typicality (**b**), and inter-response similarity (**c**) as a function of retrieval order and SVF task demands (category). Color-shaded areas depict 95% CIs of the estimated slopes

Retrieval order in the SVF task predicted a significant increase in IRT, *F*(1, 7386) = 1514.74, *p* < 0.001. This effect was reliably present for each SVF task demands level (*p* < 0.001; Fig. 1a). As expected, the main effect of the SVF task demands was significant, *F*(3, 7390) = 6.08, *p* < 0.001, such that higher task demands predicted higher IRT (see Supplementary material for more details). Furthermore, retrieval order and task demands produced a significant interaction, *F*(3, 7386) = 73.88, *p* < 0.001, indicating that the increase in IRT as a function of retrieval order was steeper in more demanding categories. Pairwise comparisons showed that the Animals (low-demand) category had the lowest IRT increase slope (*p*_Tukey_ < 0.001) from all other categories, but the slopes of other categories were not significantly different from each other (Table 1). In summary, IRT increased as a function of retrieval order and task demands.

**Table 1:** Estimated slopes of SVF task measures for each category as a function of retrieval order

Category (SVF task demands)	Semantic verbal fluency task measure		
	Inter-response time	Response typicality	Inter-response similarity
	β [95% CI]	β [95% CI]	β [95% CI]
Animals (low)	2.16 [1.81; 2.52]	− 0.50 [− 0.55; − 0.47]	− 1.82 [− 2.23; − 1.41]
Vegetables (moderate)	5.99 [5.50; 6.47]	− 0.38 [− 0.46; − 0.31]	− 2.79 [− 3.37; − 2.21]
Tools (moderate–high)	5.29 [4.77; 5.81]	− 0.69 [− 0.77; − 0.62]	− 2.73 [− 3.35; − 2.11]
Liquids (high)	5.68 [5.13; 6.22]	− 0.68 [− 0.76; − 0.60]	− 1.48 [− 2.13; − 0.82]

Inter-response time in seconds. Response typicality in logFreq. Inter-response similarity in logDice

### Effect of retrieval order and SVF task demands on response typicality

Typicality of responses gradually decreased as a function of retrieval order, *F*(1, 7389) = 969.46, *p* < 0.001, which was evident for all SVF task demands levels (*p* < 0.001; Fig. 1b). There was also a robust effect of the task demands (*p* < 0.001) and its interaction with the retrieval order (*p* < 0.001), but both effects were likely due to a narrow set of unique responses in the Vegetables category (*N* = 78, relative to *N* > 119 in the rest of the categories) (Fig. 1b light blue line). Nonetheless, even after the exclusion of the Vegetables category from the analysis, we observed a main effect of the task demands, *F*(2, 5750) = 5.38, *p* = 0.005 (although the remaining categories were not significantly different in pairwise comparisons; see Supplementary material for more details), as well as its interaction with the retrieval order, *F*(2, 5740) = 10.67, *p* < 0.001. The pairwise comparisons of the slopes indicated that in the Tools and Liquids categories the decrease in the response typicality throughout the performance was steeper compared to the Animals and Vegetables categories (*p*_Tukey_ < 0.001). However, typicality decrease slopes were not significantly different between the Tools and Liquids categories (*p*_Tukey_ = 0.998) and between the Animals and Vegetables categories (*p*_Tukey_ = 0.059), respectively (Table 1). Thus, response typicality decreased across the performance, particularly under the high task demands. However, on average, the response typicality was comparable across the SVF task demands (with the exclusion of the Vegetables category).

### Effect of retrieval order and SVF task demands on inter-response similarity

Inter-response similarity also significantly decreased as a function of retrieval order, *F*(1, 7061) = 227.48, *p* < 0.001, and this effect was present in all SVF task demands levels (*p* < 0.001; Fig. 1c). Task demands yielded a significant effect on IRS (*p* < 0.001) and interaction with the retrieval order (*p* = 0.002). Again, to ensure that these effects were not driven solely by elevated IRS in the Vegetables category (Fig. 1 c), we repeated the analysis without this category. The main effect of task demands was no longer statistically significant (*p* = 0.095, but IRS in the Animals was significantly higher than in the Tools and Liquids categories; see Supplementary material), however, its interaction with retrieval order remained significant, *F*(2, 5489) = 4.10, *p* = 0.017. Post hoc comparisons indicated that IRS had a steeper decrease across the performance in Vegetables than in Animals and Liquids categories and in the Tools compared to Liquids category (*p*_Tukey_ < 0.038). The IRS decrease slopes were not significantly different between Vegetables and Tools categories (*p*_Tukey_ = 0.816) and between Liquids and Animals categories (*p*_Tukey_ = 0.816), respectively (Table 1). Collectively, the associative similarity between consecutive responses declined throughout the performance and this decline was more evident in higher task demands (except for the Liquids category).

### Indirect effects of retrieval order on IRT trough TYP and IRS

Previous analyses confirmed the expected effect of retrieval order on IRT, TYP, and IRS. Overall, decreases in TYP (${\uprho }_{pb}$ = − 0.175, *p* < 0.001) and IRS (${\uprho }_{pb}$ = − 0.376, *p* < 0.001) significantly predicted the increase in IRT. These effects were comparable across individual SVF task demands levels (see Supplementary materials for more details).

The mediation analyses revealed that the effect of retrieval order on IRT was significantly mediated via both (decreased) TYP and IRS, with the latter showing a substantially larger proportion of the mediated effect (Table 2). In category-specific analyses (see Supplementary material for full report), the indirect effects of TYP mostly resembled the one observed across the categories in terms of mediated proportion, except for the Liquids category for which there was no significant mediation. IRS significantly mediated the increase in IRT in all categories, most notably in the Animals and Tools categories.

**Table 2:** Unstandardized parameter estimates for the mediation models for IRT via TYP and IRS

Effect_(path)_	Response typicality			Inter-response similarity		
	Estimate	95% CI	*p*	Estimate	95% CI	*p*
Total_(RO × TD + TYP|IRS → IRT)_	4.240	[4.01; 4.46]	< 0.001	4.301	[4.05; 4.54]	< 0.001
Indirect_(RO × TD → TYP|IRS → IRT)_	0.206	[0.12; 0.29]	< 0.001	0.602	[0.52; 0.69]	< 0.001
Direct_(RO × TD → IRT)_	4.034	[3.79; 4.28]	< 0.001	3.699	[3.46; 3.94]	< 0.001
Proportion mediated	0.048	[0.029; 0.070]	< 0.001	0.140	[0.121; 0.160]	< 0.001

Lower and upper confidence intervals of estimates were obtained by quasi-Bayesian approximation (5000 Monte Carlo draws)

*RO* retrieval order, *TD* SVF task demands, *TYP* response typicality, *IRS* inter-response similarity, *IRT* inter-response time

### Moderation of retrieval order’s effect on IRT by executive functioning

**Fig. 2:**
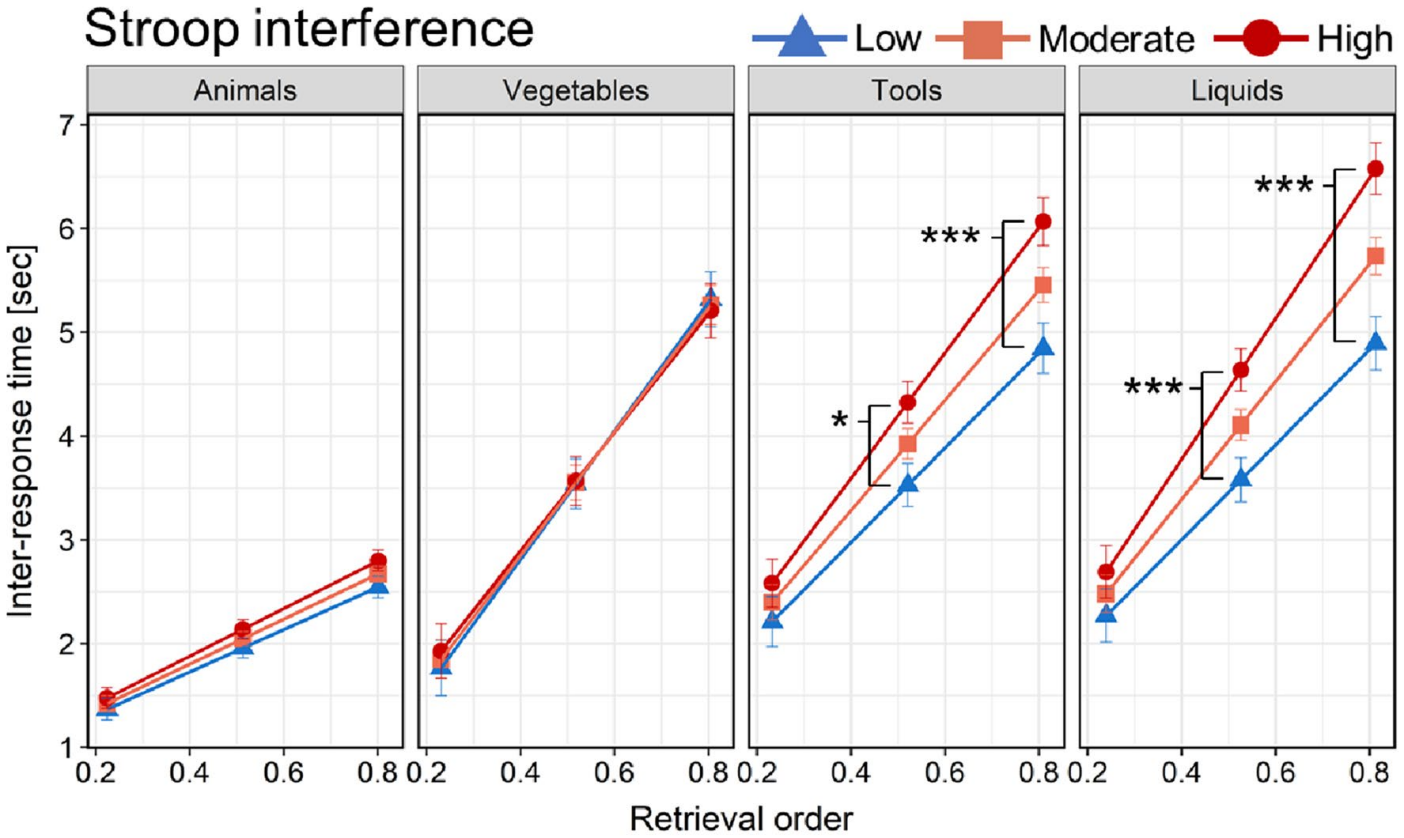
Moderated effect of retrieval order on IRT by Stroop interference across SVF task demands (categories). Note: The slopes of the retrieval order were estimated at three levels (low: − 1 SD, moderate: mean, and high: + 1 SD) of the moderator. *p < .05; ***p < .001 (differences in IRT at distinct retrieval order positions between the levels of the moderator; adjusted by Holm–Bonferroni correction). Error-bars show ± SE

A significant three-way interaction between the retrieval order, task demands, and Stroop interference on IRT was observed, *F*(3, 7379) = 5.711, *p* < 0.001. We found no evidence for the moderation of retrieval order effect on IRT by Stroop interference in low (Animals) and moderate (Vegetables) task demands (both *p*_Holm_ = 0.456). Conversely, in the moderate-high (Tools), *F*(1, 1359) = 6.48, *p*_Holm_ = 0.033, and high (Liquids) task demands, *F*(1, 1255) = 9.34, *p*_Holm_ = 0.008, the Stroop interference significantly moderated the effect of the retrieval order on IRT, such that the effect scaled up with the amount of measured Stroop interference (Table 3 and Fig. 2).

**Table 3:** Conditional effects of the predictor (retrieval order) on IRT at different levels of the moderator (Stroop interference)

SVF task demands	Moderation	β	*SE*	*t*	*p*_Holm_
Low (Animals)	Retrieval order × Stroop interference	0.909	0.755	1.205	0.456
Moderate (Vegetables)	Retrieval order × Stroop interference	− 1.744	2.067	− 0.844	0.456
Moderate–high (Tools)	Retrieval order × Stroop interference	5.341	2.099	2.545	0.033
	Low level of Stroop interference (93)	4.562	0.412	11.082	< 0.001
	Moderate level of Stroop interference (231)	5.299	0.292	18.174	< 0.001
	High level of Stroop interference (369)	6.037	0.411	14.705	< 0.001
High (Liquids)	Retrieval order × Stroop interference	7.824	2.560	3.056	0.008
	Low level of Stroop interference (90)	4.566	0.508	8.990	< 0.001
	Moderate level of Stroop interference (230)	5.666	0.358	15.805	< 0.001
	High level of Stroop interference (371)	6.766	0.508	13.315	< 0.001

The values in the brackets for the respective levels of the moderator represent the measured Stroop interference (in ms) at chosen points of the sample distribution (− 1 SD, mean, and + 1 SD) upon which the conditional slopes were estimated

**Fig. 3:**
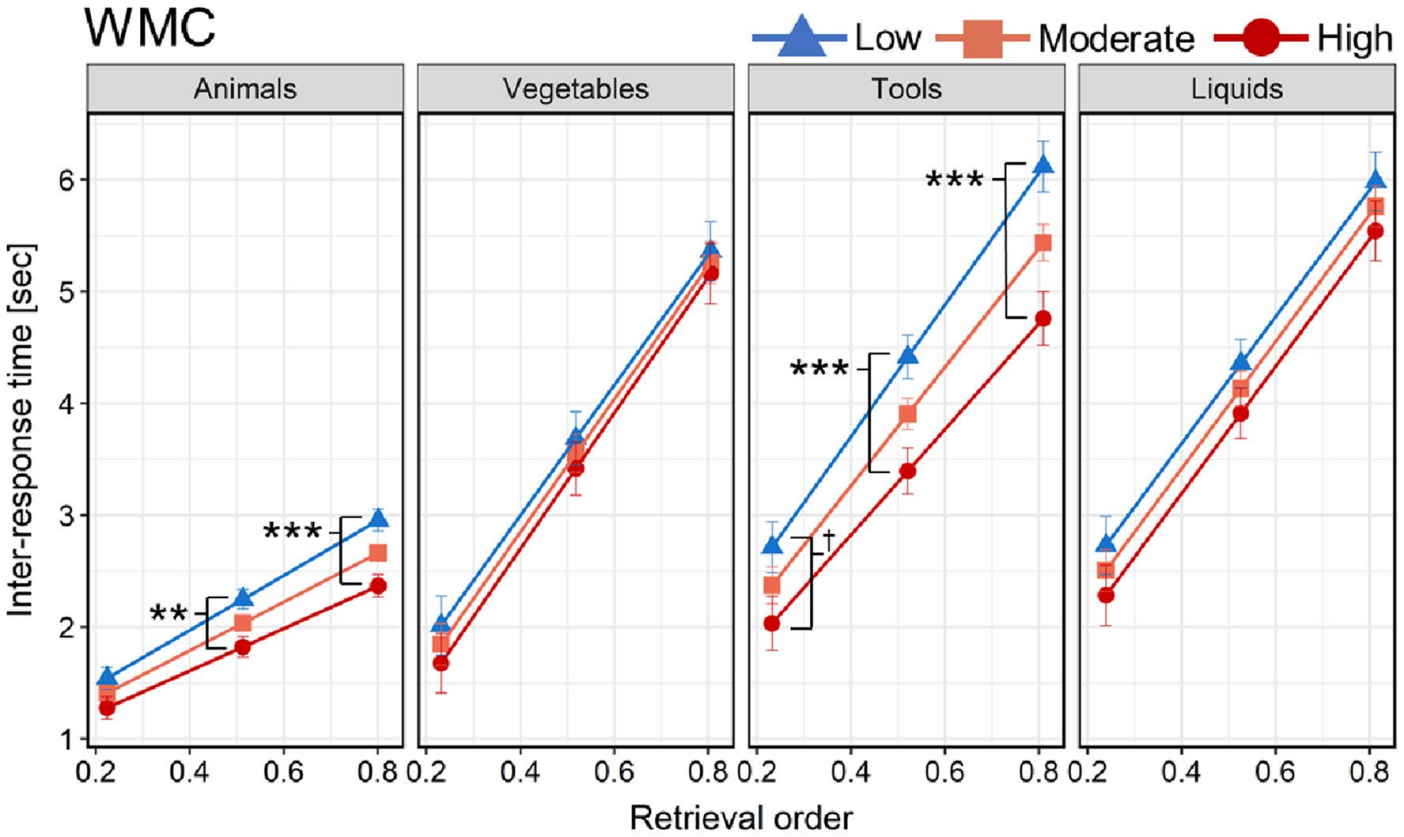
Moderated effect of retrieval order on IRT by working memory capacity across SVF task demands (categories). Note: The slopes of the retrieval order were estimated at three levels (low: − 1 SD, moderate: mean, and high: + 1 SD) of the moderator. ^†^*p* < .10; **p < .01; ***p < .001 (differences in IRT at distinct retrieval order positions between the levels of the moderator; adjusted by Holm–Bonferroni correction). Error-bars show ± SE

There was no significant interaction between retrieval order, task demands, and WMC on IRT, *F*(3, 7379) = 1.494, *p* = 0.214. However, WMC significantly moderated the effect of retrieval order on IRT in low (Animals) SVF task demands, *F*(1, 2951) = 7.24, *p*_Holm_ = 0.028, such that the effect of retrieval order on IRT scaled down with higher WMC (Table 4 and Fig. 3). A similar trend for such moderation was also observed in the moderate–high (Tools) task demands, but the effect was significant only before the correction for multiple tests, *F*(1, 1358) = 4.09, *p*_Holm_ = 0.129 (before the correction *p* = 0.043) (Fig. 3). WMC did not moderate the effect of retrieval order on IRT in the rest of the SVF task categories (*p*_Holm_ > 0.999).

**Table 4:** Conditional effects of the predictor (retrieval order) on IRT at different levels of the moderator (working memory capacity)

SVF task demands	Moderation	β	*SE*	*t*	*p*_Holm_
Lower (Animals)	Retrieval order × WMC	− 0.334	0.124	− 2.691	0.028
	Low level of WMC (4.03)	2.449	0.147	16.669	< 0.001
	Moderate level of WMC (4.87)	2.170	0.104	20.911	< 0.001
	High level of WMC (5.71)	1.890	0.147	12.894	< 0.001
Moderate (Vegetables)	Retrieval order × WMC	0.144	0.342	0.421	> 0.999
Moderate-high (Tools)	Retrieval order × WMC	− 0.689	0.341	− 2.022	0.129
High (Liquids)	Retrieval order × WMC	0.007	0.430	0.017	> 0.999

The values in the brackets for the respective levels of the moderator represent the measured average span in the backward digit span task at chosen points of the sample distribution (− 1 SD, mean, and + 1 SD) upon which the conditional slopes were estimated

SCT and ACT measures did not significantly interact with the retrieval order and task demands, *F* < 1.968, *p* > 0.117. Specifically, controlled search and selective demands cost measures from the SCT did not moderate the retrieval order’s effect on IRT in neither of the task demands (*p*_Holm_ > 0.276 and *p*_Holm_ > 0.328, respectively). For the inhibition cost measure from the ACT, a trend for moderation was observed in the moderate (Vegetables) task demands, *F*(1, 1554) = 5.40, β = 0.546, SE = 0.235, *p*_Holm_ = 0.080 (before the correction *p* = 0.020), showing that the retrieval order’s effect on IRT scaled up with the amount of the measured inhibition cost (low level: 0.592 s, β = 5.274, SE = 0.406; moderate level: 1.818 s, β = 5.944, SE = 0.288; high level: 3.045 s, β = 6.613, SE = 0.288, all *p* < 0.001). However, the inhibition cost was not a significant moderator of the retrieval order effect on IRT in the rest of the SVF task demands (*p*_Holm_ > 0.999). Likewise, we found no moderation effect of the switching cost measure from the ACT in any of the SVF task demands (*p*_Holm_ > 0.732). Therefore, while interference control moderated the amount of retrieval slowing especially in high-demand categories, working memory effect seemed to apply both in low and partially in more demanding categories. Notably, semantic control capacities did not show significant moderating effects.

## Discussion

The current views (e.g., Hills et al., 2013; Mayr & Kliegl, 2000; Troyer et al., 1997) put little emphasis on how the automatic and controlled processes relate to the SVF task performance over time. Recent research (Broday-Dvir & Malach, 2021; Demetriou & Holtzer, 2017; Katzev et al., 2013), however, indicates that the extent to which SVF task performance may be predicted by executive functioning is time-dependent. That is, as the typicality or connectedness of probed information in semantic memory deteriorates with continuous performance, retrieval may become less spontaneous (automatic) and more executively demanding (controlled). Extending these suggestions, we assessed how temporal changes in these semantic attributes affect the retrieval fluency throughout the continuous performance in the SVF task. Furthermore, we tested the prediction that individual differences in executive control capacity moderate the slowing of retrieval fluency across the task performance, particularly in less organized (i.e., high demand) semantic categories.

In line with our assumptions, we found that retrieval fluency gradually decreased with the response retrieval order. Moreover, later responses were not only slower but also less category-typical and inter-connected, which corresponds with previous findings (Beaty & Silvia, 2012; Crowe, 1998; Linz et al., 2019). Furthermore, the retrieval slowing was more pronounced under higher SVF task demands, suggesting that gradually increasing associative sparseness could impact semantic retrieval more severely in poorly organized categories (Mayr & Kliegl, 2000). In support of this notion, our correlation analysis revealed that retrieval latency was longer for responses that were both less category-typical and less strongly semantically related. Yet, the subsequent mediation analyses showed that the decrease in semantic similarity compared to response typicality explained a considerably larger proportion of the time-dependent prolongation of retrieval latency. This indicates that the dynamics of semantic retrieval is determined more strongly by the degree of relatedness to previously recalled items than by representational availability (Hills et al., 2015; Thompson & Kello, 2014). Together, these findings support the assumption that spreading activation dissipates as associative links between category exemplars become weaker, indicating that the efficacy of automatic retrieval decreases throughout the performance, which is in line with our hypothesis.

Considering the contribution of the controlled processing, only domain-general, but not domain-specific, executive capacities reliably moderated the effect of retrieval order on retrieval fluency. Worse interference control was associated with greater retrieval slowing throughout the performance, particularly in the categories with higher task demands, showing that as retrieval progresses from earlier to later instances, it might become more executively driven, which further supports our hypothesis. The finding that high-demand categories evoked more overt intrusions across response sequences complementarily bolsters the idea that interference control may be more taxed in later SVF performance, particularly under the conditions of restricted response options and severe intrusions (Henry & Crawford, 2004; Raboutet et al., 2010; Rosen & Engle, 1997). Interestingly, the moderation by WMC did not uniquely pertain to high-demand SVF task categories. Instead, the moderations were evidenced in the low-demand and partially in the moderate-high demand category. In both cases, poor WMC predicted steeper retrieval slowing over the performance. Overall, these results align with the proposed role of working memory, which maintains and updates semantic cues to guide the retrieval process, and indicate its importance in keeping the retrieval fluent when progressing from readily to less accessible category exemplars (Hills et al., 2013; Unsworth & Engle, 2007; Unsworth et al., 2011). Moreover, these category-specific moderations may reflect other topological properties of the given categories that could motivate greater recruitment of working memory into semantic retrieval. In particular, the ability to self-generate retrieval cues to probe category exemplars might prove more advantageous when the exemplars are sorted into distinct semantic patches rather than loosely organized (Hills et al., 2013; Troyer et al., 1998; Unsworth et al., 2013). Future studies should investigate whether semantic categories with patchy semantic structure selectively predict better SVF task performance related to higher WMC. Nonetheless, the current findings indicate that preservation of fluent retrieval across time in the SVF task is primarily supported by interference control and WMC. While the first resolves the interference brought by typical and densely inter-connected concepts that are not appropriate or have been already retrieved, the latter is required for effective maintenance and updating of retrieval cues or strategies (Rosen & Engle, 1997; Unsworth et al., 2013).

Noteworthy in this sense are the views (Abbott et al., 2012, 2015) arguing that fluency behavior can be accounted for by a single search process, i.e., a random-walk able to produce phenomena akin to swift or slower transitions between highly versus less similar items, intrusions or repetitions, when applied to the semantic network. This position would promote the idea that structural properties of semantic memory alone may suffice to explain variability across the SVF task performance. In agreement, our results show that a vast portion of retrieval performance variability pertains to changes in typicality and similarity of the category exemplars (i.e., structural properties). Yet, we have also shown that individual ability to withhold relevant information and forfeit task-irrelevant information relates to how these structural properties impact the retrieval process. Relatedly, other semantic retrieval paradigms comply with this notion. For example, an established view in the research of semantic priming claims that working memory facilitates lexical access by generating retrieval targets based on presented retrieval cues (or primes) (e.g., Neely et al., 1989).

Taken together, our data strongly suggest that the gradual slowing of retrieval in the SVF task due to the depletion of highly typical and inter-connected category exemplars could be compensated by the recruitment of the domain-general executive processes. This account is consistent with the view that verbal fluency benefits from executive updating and monitoring functions that mediate effective access to semantic knowledge (Rosen & Engle, 1997; Unsworth & Engle, 2007) and also with the observations that verbal fluency is affected by frontal lobe dysfunction (Henry & Crawford, 2004; Tröster et al., 1998).

﻿The lack of evidence for an association between verbal fluency and (a) controlled semantic search and selective demands costs in SCT and (b) inhibition and switching costs in ACT, on the other hand, indicates that the semantic control capacity might be less important to counteract associative sparsity in later SVF task performance. The reason for this might result from two specific characteristics of the SVF task. First, since the retrieval in SVF tends to adhere to temporally-optimized information foraging (Abbott et al., 2015; Hills et al., 2015), the strict time constraints of the task could have curtailed a thorough search for weakly associated concepts, which is mainly supported by the semantic control system (Badre & Wagner, 2007). Second, the fact that SVF involves retrieval from within a specific category naturally predisposes the responses to be associatively related, thus decreasing the need for substantial representational shifts (switching) among associatively distant concepts (Badre & Wagner, 2007; Chiou et al., 2018) or the need to completely suppress (inhibit) the active semantic set, as required in the ACT (Marko & Riečanský, 2021; Marko et al., 2019a; Marko et al., 2019b). Notably, we do not argue that semantic retrieval does not generally benefit from these processes, but only that such processes may not be substantially engaged in the SVF task. Instead, the category constraint in the SVF task could more likely lead to cumulative semantic interference induced from typical or prepotent category exemplars, especially in the later performance, which would result in the engagement of a domain-general control network that selectively gates the relevant semantic knowledge (Oppenheim et al., 2010). For example, activity in the left inferior frontal gyrus (BA 45) has been documented across SVF tasks using categories of both low and high demands (Costafreda et al., 2006; Hirshorn & Thompson-Schill, 2006), but also to increase when retrieving from high-demand categories (Katzev et al., 2013). This region is also functionally linked with the retention of semantic representations in working memory (Fiebach et al., 2007) and with the resolution of semantic interference (Liu et al., 2015; Luo, 1999). Therefore, it is plausible to assume that frontally driven controlled retrieval is mediated by different capacities across SVF task demands to facilitate retrieval in later performance (i.e., working memory facilitates the later performance mainly in lower demand categories with patchy semantic structure, while interference control might be critical for later retrieval in high-demand categories with sparser organization and fewer retrieval options).

Importantly, the current results also point out that commonly used SVF measures of clustering and switching may not accurately reflect how automatic and controlled processes engage during category-cued memory retrieval. Specifically, the coupling between weakening relatedness and retrieval slowing shows that SVF cannot be sufficiently described as alternations between rapid bursts of strongly associated concepts and slower switches initiating new subcategories (Troyer et al., 1998). Moreover, should we assume that switching during retrieval exerts increased demands on executive attention (Hirshorn & Thompson-Schill, 2006), our findings then indicate that these demands vary in time and across categories. This suggests that switches occurring early in the response sequence are less demanding, while later switches (or even clustering) may require greater retrieval control. With these regards, our findings support the notion that the assessment of SVF in neurotypical individuals and patients may benefit from acquiring fluency measures at distinct retrieval positions (or stages of the performance). Whereas poorer performance at the initial phases of the SVF task might primarily indicate degraded semantic knowledge (e.g., in semantic dementia; Henry et al., 2004; Martin & Fedio, 1983), worse performance in the SVF task during later phases might specifically point to compromised executive functioning (e.g., in dysexecutive syndrome or mild cognitive impairment; Blanco Martín et al., 2016; Demetriou & Holtzer, 2017).

Finally, this study has several limitations that need to be addressed. First, it remains an open question whether the measures of response similarity and typicality are sensitive enough to detect subtle changes in associative structure throughout the task, especially given that the range of semantic distances among responses in the SVF task is inherently constrained to a category. Therefore, it should be considered that the estimates involving these measures may represent lower bounds of the true effects. Second, the individual-differences approach and correlational design of the study limit the interpretations of the processes that might have driven the relationships between the SVF task and executive control tasks performance. Nevertheless, these effects provide a firm template for generating more elaborated experimental predictions regarding the time-dependent involvement of control processes in the SVF task. Third, we used only a single measure for each executive function when considering the main hypothesis. Assessing multiple measures of the same construct might allow for a more powerful statistical approach via latent variable modeling, potentially providing a better and more reliable approximation of the true effects (Friedman & Miyake, 2017). Forth, assessing the individual extent of knowledge (vocabulary) in future studies could be beneficial as it may be associated with the relative involvement of automatic-associative retrieval versus executive control mechanisms at various points in performance in the SVF task.

## Conclusions

In this study, we tested the hypothesis that as retrieval in the SVF task progresses from highly to less accessible semantic knowledge, the performance depends less on automatic and more on controlled executive capacities. In line with this hypothesis, our results show that (1) the continuous retrieval slowing in the SVF task is characterized by lower typicality and semantic relatedness of the category exemplars and (2) that individual differences in interference control play role in this retrieval slowing, particularly in high-demand SVF task categories. These findings provide convincing support for the view that retrieval during the SVF task depends on both automatic-associative and domain-general controlled processes whose contribution changes complementarily as a function of time and associative sparsity. This evidence may provide implications for the use of the SVF task in clinical assessment and yields additional insights into the nature of the processes underlying performance in this task.

### Supplementary Information

Below is the link to the electronic supplementary material.

Supplementary file1 (PDF 372 kb)

## Data Availability

The data (including English translation of SVF responses) and materials for all conducted analyses are available at OSF repository (url: http://osf.io/s63yh).
