# Correction to: Executive functioning moderates the decline of retrieval fuency in time

**DOI:** 10.1007/s00426-024-01993-2

**Published:** 2024-06-20

**Authors:** Drahomír Michalko, Martin Marko, Igor Riečanský

**Affiliations:** 1 Department of Behavioural Neuroscience, Institute of Normal and Pathological Physiology, Centre of Experimental Medicine, Slovak Academy of Sciences, Sienkiewiczova 1, Bratislava, 813 71 Slovakia; 2 Department of Applied Informatics, Faculty of Mathematics, Physics and Informatics, Comenius University in Bratislava, Mlynská Dolina F1, Bratislava, 842 48 Slovakia; 3 Social, Cognitive and Affective Neuroscience Unit, Department of Cognition, Emotion, and Methods in Psychology, Faculty of Psychology, University of Vienna, Liebiggasse 5, Vienna, 1010 Austria; 4 Department of Psychiatry, Faculty of Medicine, Slovak Medical University in Bratislava, Limbová 12, Bratislava, 833 03 Slovakia

**Psychological Research, Volume 87, pages 397?409, (2023).**

10.1007/s00426-022-01680-0

The article “Executive functioning moderates the decline of retrieval fuency in time”, written by Drahomír Michalko, Martin Marko and Igor Riečanský., was originally published Online First without Open Access. After publication in volume 87, issue 2, page 397–409 the author decided to opt for Open Choice and to make the article an Open Access publication. Therefore, the copyright of the article has been changed to © the author(s) 2024 and the article is forthwith distributed under the terms of the Creative Commons attribution 4.0 international license, which permits use, sharing, adaptation, distribution and reproduction in any medium or format, as long as you give appropriate credit to the original author(s) and the source, provide a link to the Creative Commons licence, and indicate if changes were made. The images or other third party material in this article are included in the article’s Creative Commons licence, unless indicated otherwise in a credit line to the material. If material is not included in the article’s Creative Commons licence and your intended use is not permitted by statutory regulation or exceeds the permitted use, you will need to obtain permission directly from the copyright holder. To view a copy of this licence, visit http://creativecommons.org/licenses/by/4.0.

Original article has been corrected

