# Supplementary material for: Executive functioning moderates the decline of retrieval fluency in time
Source: Psychol Res. 2022 Apr 25;87(2):397–409. doi: 10.1007/s00426-022-01680-0 (PMC11189984; doi:10.1007/s00426-022-01680-0)
Supplement: Supplementary file 1 — Supplementary file1 (PDF 372 kb) [file 426_2022_1680_MOESM1_ESM.pdf]

Supplementary material

Executive functioning moderates the decline of retrieval fluency in time

Drahomír Michalko, Martin Marko, Igor Riečanský

email: drahomir.michalko@savba.sk

Method

Evaluation rules for the SVF task responses

For *Animals* category, we excluded all exemplars that were male (or female) version of the previously mentioned species (e.g., ram in case of sheep being mentioned first). In the case of the *Vegetables* category, we excluded the variations of the same vegetable (e.g., if bell pepper was named first, then red, or yellow peppers were not included because they represented the same kind of vegetable). In the *Tools* category, we excluded mainly the slang synonyms, or variations of the same thing (e.g., screwdrivers with different heads – e.g., cross, flat). For the *Liquids* category, participants were told not to list the names of the labels (e.g., coca-cola). If this occurred, we retained the first item (name of the label) to represent for example sweet drinks or sodas. However, if other instances would occur (e.g., fanta, sprite, etc.) than these would be removed. Moreover, if participant entered a word "lake" first and then typed in other water surface areas (e.g., river, ocean), we considered it as different instances of the same thing and removed such category exemplars. Similarly, we removed instances of alcohol distillates that referred to the same type of the alcohol only made of different ingredients (e.g., out of apples, pears, plums, etc.) and retained only the first named exemplar.

Semantic control task stimulus materials

The full list of items is downloadable in English version at the OSF repository (url: osf.io/s63yh). Items belonging to the Global and Feature conditions were divided into separate blocks (four for each condition). These blocks were then organized into four super-ordinate blocks that contained one Global and one Feature condition block. These super-ordinate blocks were presented randomly, however, within them a Global block was always presented first. This ensured that participants always begun with a Global block. Condition blocks were a priori randomly paired into the super-ordinate blocks. Each block from both Global and Feature condition contained equal number of strongly and weakly related, and equal number of congruent and incongruent trials. Furthermore, in Feature blocks the trails were assembled such that each block contained roughly the same number of feature (color, shape, size, texture) types. Trials were displayed randomly within the blocks. Likewise, trials were assigned to individual blocks on random basis (random.org)

Regarding the lexical properties of the items, we compared the stimuli in word frequency, length, and semantic relatedness that was operationalized by the logDice measure derived from the national corpus database. For the Global condition, the cue, target, and alternative words did not differ significantly between strong and weak trials in word frequency and word length, $ts(30) < 1.62$, $ps > .116$. However, in strong trials, the target words were significantly more related to the cue words than in the weak trials, $t(30) = 30.72$, $p < .001$. Alternative words were not differently related to the cue words in a significant manner, $t(30) = -1.00$, $p = .325$. Moreover, for both strong and weak trials, target and alternative words did not differ significantly in word frequency and length, $ts(30) < 1.55$, $ps > .131$

In the Feature condition, cue words in congruent trials had statistically higher word frequency compared to incongruent trials, [$t(30) = 3.22$, $p = .003$], but did not differ in mean word length, $t(30) = -0.26$, $p = .796$. Target and distractor (alternative) words were also not statistically different in word frequency and length, [$ts(30) < 1.31$, $ps > .199$] between congruent and incongruent trials. However, the targets were significantly more semantically related to the cue words in the congruent than in incongruent trials, $t(30) = 30.03$, $p < .001$. Conversely, the distractor words were significantly more related to the cue words in the incongruent compared to congruent trials, $t(30) = -35.75$, $p < .001$. Similarly, in both congruent and incongruent trials, target and alternative words did not differ significantly in word frequency and length, $ts(30) < -1.34$, $ps > .190$. The procedure with example trials is depicted in the Figure S1.

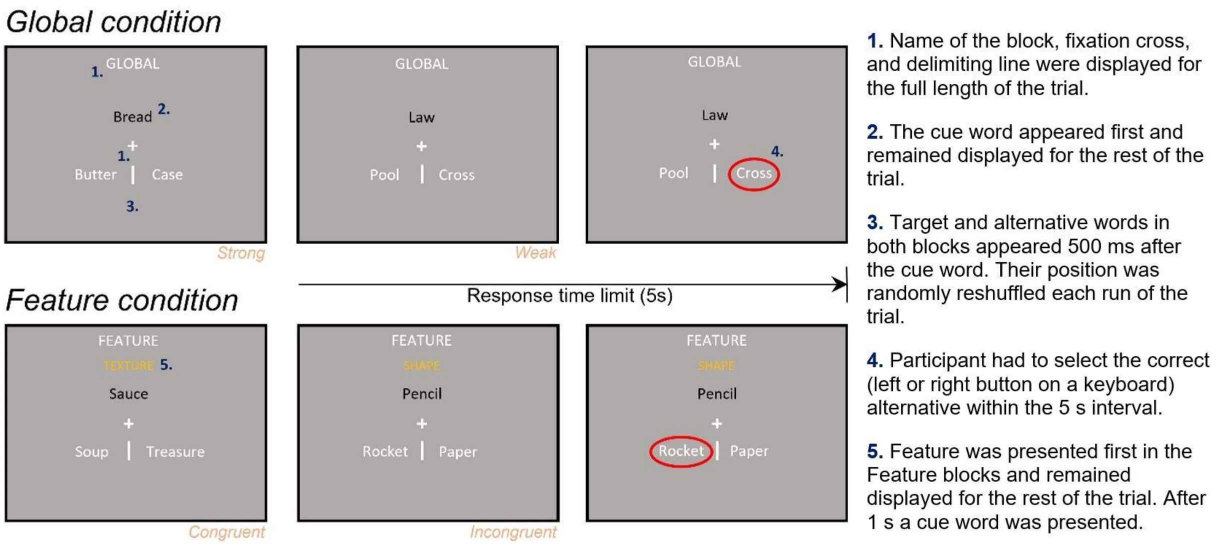

Fig. S1 SCT procedure and example trials for Global and Feature conditions

Data processing

The SVF task response appropriateness was evaluated by three independent raters (absolute agreement average-measure *ICC* = .937). Following this procedure, responses judged as inappropriate (5.40%), extremely slow responses (IRI > 35s; 0 .44%), and repetitions (2.01 %) were excluded. The remaining IRT data were winsorized (10% two-sided trimming) across participants and SVF task demands (i.e., individual categories). In the Stroop task data, erroneous trials (1.89%) and trials with RT > 3 *SD* (1.62%) were excluded before computing individuals' averages. In the SCT data, four test items from the feature-incongruent condition had > 60% error rate and hence were excluded from further analyses. Additionally, we removed erroneous responses (7.77%) and responses with RT > 3 *SD* (0.64%; across all matching rule × trial types) before computing individual contrasts. In ACT data, responses that either did not comply with the retrieval rule or took > 20 sec were excluded (8.02%), following which the remaining RTs were winsorized (10% two-sided trimming) across participants, retrieval type (associative, dissociative) and chain type (fixed, alternating) conditions. Note that the final RT contrasts from executive control measures were also winsorized (10% two-sided trimming).

Results

Contrasts from executive control tasks

The data from the Stroop task showed a large and significant interference effect with longer RTs in incongruent trials (*M* = 1002; 95%CI [960; 1044]) than in congruent trials (*M* = 753; CI [727; 779]) with the mean difference of 249ms [215; 284], $t(84) = 14.32$, $p < .001$, Cohen's $d = 1.55$.

In the SCT, comparison of weakly related (*M* = 2.23; CI [2.16; 2.29]) and strongly related trials (*M* = 1.35; CI [1.29; 1.41]) in the Global condition revealed a robust controlled semantic search cost with the mean difference of 0.87s [0.84; 0.91], $t(84) = 46.33$, $p < .001$, Cohen's $d = 5.03$. Similarly, longer RTs in incongruent (*M* = 2.09; CI [2.01; 2.17) compared to congruent trials (*M* = 1.74; CI [1.67; 1.82]) in the Feature condition indicated a significant selective demands cost with the mean difference of 0.35s [0.30; 0.40], $t(84) = 14.32$, $p < .001$, Cohen's $d = 1.55$.

Finally, in the ACT, the comparison between associative chain RT (*M* = 4.14; CI [3.76; 4.53]) and dissociative chain RT (*M* = 5.90; CI [5.51; 6.29]) revealed a large and significant inhibition cost with the mean difference of 1.75s [1.55; 1.96], $t(8005) = 16.94$, $p < .001$, Cohen's $d = 1.35$. Moreover, the retrieval type and chain type factors produced a significant interaction, $F(1, 8003) = 49.60$, $p < .001$. There was no difference between the associative RT in fixed (*M* = 4.14; CI [3.76; 4.53]) and the associative RT in alternating chain (*M* = 4.27; CI [3.88; 4.66]), $t(8003) = 1.25$, $p = .596$. However, dissociative RT was longer in alternating (*M* = 7.06; CI [6.67; 7.45]) compared to fixed chain type (*M* = 5.90; CI [5.51; 6.29]), confirming a significant switching cost with the mean difference of 1.16s [0.95; 1.37]), $t(8004) = 10.93$, $p < .001$, Cohen's $d = 1.21$.

Main effect of the SVF task demands on IRT (pairwise comparisons)

On average, the lowest IRT was in the low demands (Animals; *M* = 2.09; CI [1.88; 2.29]) compared to all other SVF task demands levels, $t > 15.668$, $p_{\text{Tukey}} < .001$. Moreover, the IRT in the moderately demanding category (Vegetables; *M* = 3.48; CI [3.26; 3.70]) was significantly lower compared to moderately-highly (Tools; *M* = 3.90; CI [3.67; 4.13]) and highly demanding categories (Liquids; *M* = 4.11; CI [3.88; 4.34]), $t > 4.015$, $p_{\text{Tukey}} < .001$. However, the IRT was not significantly different between the moderate-high and high SVF task demands, $t = 1.902$, $p_{\text{Tukey}} = .227$.

Main effect of the SVF task demands on intrusions

Task demands had a moderate and significant main effect on the number of intrusions in a response sequence, $F(3, 252) = 14.651$, $p < .001$, $\eta^2 = .149$, showing that intrusions occurred more often in high demand than low demand categories. Pairwise comparisons indicated highest intrusion rate in the Liquids category compared to all other categories ($p_{\text{Holm}} < .007$). Tools category produced significantly larger proportion of intrusions than Animals and Vegetables category ($p_{\text{Holm}} < .049$) (Figure S2).

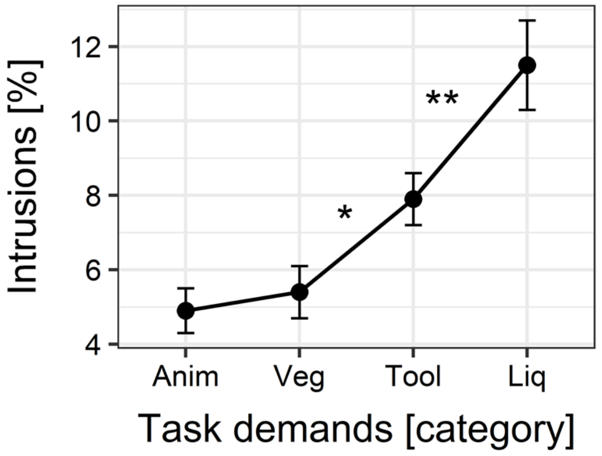

Fig. S2 Proportion of intrusions (i.e., inappropriate words, synonyms, or repetitions) across SVF response sequences as a function of task demands. ** $p < .01$ * $p < .05$ (Holm correction).

Main effect of the SVF task demands on TYP (pairwise comparisons)

Despite a significant effect of the task demands (without the Vegetables category) on TYP, we did not observe any significant differences between the respective task demands levels in terms of average TYP, $t < 1.599$, $p_{\text{Tukey}} = .246$.

Main effect of the SVF task demands on IRS (pairwise comparisons)

On average, the IRS in the low (Animals; $M = 4.62$; CI [4.49; 4.75]) task demands was significantly higher than in moderate-high (Tools; $M = 4.11$; CI [3.93; 4.29]) and high (Liquids; $M = 4.29$; CI [4.10; 4.48]) task demands, $t > 3.020$, $p_{\text{Tukey}} < .007$. Moderate-high and high task demands did not produce responses with significantly different IRS, $t = 1.460$, $p_{\text{Tukey}} = .311$.

Category specific mediations

Correlations of TYP and IRS with IRI. Lower TYP predicted longer IRT in the Animals (ρ_{pb} = -.184, $p < .001$), Vegetables (ρ_{pb} = -.204, $p < .001$), Tools (ρ_{pb} = -.214, $p < .001$) and Liquids (ρ_{pb} = -.219, $p < .001$) category. Similarly, lower IRS prolonged IRT in Animals (ρ_{pb} = -.443, $p < .001$), Vegetables (ρ_{pb} = -.374, $p < .001$), Tools (ρ_{pb} = -.418, $p < .001$), and Liquids (ρ_{pb} = -.382, $p < .001$) categories. See Tables S1 – S4 for the results of category-specific mediation analyses.

Table S1. *Unstandardized parameter estimates for the mediation models for IRT via TYP and IRS (Animals category)*

**Effect$_{(path)}$**	**Response typicality**			**Inter-response similarity**		
	Estimate	95% CI	*p*	Estimate	95 % CI	*p*
Total$_{(RO \times TD + TYP \mid IRS \rightarrow IRT)}$	2.169	[1.96; 2.37]	< .001	2.192	[1.98; 2.41]	< .001
Indirect$_{(RO \times TD \rightarrow TYP \mid IRS \rightarrow IRT)}$	0.175	[0.10; 0.25]	< .001	0.382	[0.29; 0.47]	< .001
Direct$_{(RO \times TD \rightarrow IRT)}$	1.995	[1.77; 2.22]	< .001	1.811	[1.61; 2.02]	< .001
Proportion mediated	.080	[.047; .120]	< .001	.174	[.137; .210]	< .001

Note. Lower and upper confidence intervals of estimates were obtained by quasi-Bayesian approximation (5000 Monte Carlo draws). RO – retrieval order; TD – SVF task demands; TYP – response typicality; IRS – inter-response similarity; IRT – inter-response time.

Table S2. *Unstandardized parameter estimates for the mediation models for IRT via TYP and IRS (Vegetables category)*

**Effect$_{(path)}$**	**Response typicality**			**Inter-response similarity**		
	Estimate	95% CI	*p*	Estimate	95 % CI	*p*
Total$_{(RO \times TD + TYP \mid IRS \rightarrow IRT)}$	5.942	[5.36; 6.50]	< .001	6.239	[5.61; 6.86]	< .001
Indirect$_{(RO \times TD \rightarrow TYP \mid IRS \rightarrow IRT)}$	0.233	[0.04; 0.43]	.021	0.803	[0.59; 1.03]	< .001
Direct$_{(RO \times TD \rightarrow IRT)}$	5.709	[5.11; 6.31]	< .001	5.435	[4.81; 6.05]	< .001
Proportion mediated	.039	[.007; .070]	.021	.128	[.096; .170]	< .001

Note. Lower and upper confidence intervals of estimates were obtained by quasi-Bayesian approximation (5000 Monte Carlo draws). RO – retrieval order; TD – SVF task demands; TYP – response typicality; IRS – inter-response similarity; IRT – inter-response time.

Table S3. *Unstandardized parameter estimates for the mediation models for IRT via TYP and IRS (Tools category)*

**Effect$_{(path)}$**	**Response typicality**			**Inter-response similarity**		
	Estimate	95% CI	*p*	Estimate	95 % CI	*p*
Total$_{(RO \times TD + TYP \mid IRS \rightarrow IRT)}$	5.311	[4.74; 5.60]	< .001	5.350	[4.72; 5.97]	< .001
Indirect$_{(RO \times TD \rightarrow TYP \mid IRS \rightarrow IRT)}$	0.319	[0.08; 0.56]	.005	1.001	[0.74; 1.28]	< .001
Direct$_{(RO \times TD \rightarrow IRT)}$	4.992	[4.37; 5.60]	< .001	4.349	[3.76; 4.95]	< .001
Proportion mediated	.060	[.016; .110]	.005	.186	[.141; .240]	< .001

Note. Lower and upper confidence intervals of estimates were obtained by quasi-Bayesian approximation (5000 Monte Carlo draws). RO – retrieval order; TD – SVF task demands; TYP – response typicality; IRS – inter-response similarity; IRT – inter-response time.

Table S4. *Unstandardized parameter estimates for the mediation models for IRT via TYP and IRS (Liquids category)*

**Effect$_{(path)}$**	**Response typicality**			**Inter-response similarity**		
	Estimate	95% CI	*p*	Estimate	95 % CI	*p*
Total$_{(RO \times TD + TYP|IRS \rightarrow IRT)}$	5.676	[4.97; 6.40]	< .001	5.590	[4.79; 6.38]	< .001
Indirect$_{(RO \times TD \rightarrow TYP|IRS \rightarrow IRT)}$	0.206	[-0.08; 0.49]	.160	0.605	[0.32; 0.90]	< .001
Direct$_{(RO \times TD \rightarrow IRT)}$	5.469	[4.70; 6.25]	< .001	4.985	[4.22; 5.74]	< .001
Proportion mediated	.036	[.000; .090]	.160	.108	[.059; .160]	< .001

Note. Lower and upper confidence intervals of estimates were obtained by quasi-Bayesian approximation (5000 Monte Carlo draws). RO – retrieval order; TD – SVF task demands; TYP – response typicality; IRS – inter-response similarity; IRT – inter-response time.

The effect of task order on SVF task performance

Since the tasks were administered in a random order, it can be suspected that prior performance on other semantic tasks (ACT and SCT) could affect IRT in the SVF task (i.e., some stimuli used in the ACT or SCT task may have increased the availability of certain items in the memory, making a subsequent performance in the SVF task less demanding). Therefore, we evaluated whether preceeding ACT or SCT performance affected IRT in the SVF task. We created three grouping variables assigning participants either to a group where ACT or SCT preceeded the SVF task (n = 54), only ACT preceeded the SVF (n = 37), or only the SCT preceeded the SVF (n = 41). Effects of these grouping variables were tested by three separate LMEMs that also included SVF task category factor to examine whether such effects apply only to certain semantic categories.

We found no significant difference in IRT when both ACT and/or SCT preceeded versus followed the SVF task, $F(1, 85) = 0.749$, $p = .389$, nor the interaction with the SVF task category, $F(3, 7403) = 0.745$, $p = .525$. The task order also had no main effect on the IRT when only the ACT preceeded the SVF task, $F(1, 84) = 1.047$, $p = .309$, nor it interacted with the SVF task category, $F(3, 7405) = 2.410$, $p = .065$. Pairwise contrasts did not reveal any significant differences in IRT ($p_{Tukey} > .643$). Finally, the task order had no significant main effect on the IRT when only the SCT preceeded the SVF task, $F(1, 84) = 0.053$, $p = .818$ and likewise did not interact with the SVF task category factor, $F(3, 7404) = 0.938$, $p = .421$. These analyses show that order of the semantic tasks did not significantly influence the target IRT variable in the SVF task.
